# Using impedance-based approaches for measuring cell-mediated cytotoxicity and antibody-dependent cell-mediated cytotoxicity (ADCC)

**DOI:** 10.1186/2051-1426-3-S2-P214

**Published:** 2015-11-04

**Authors:** Brandon Lamarche, Yama Abassi

**Affiliations:** 1 ACEA Biosciences, San Diego, CA, USA

## 

The most common method for measuring cell-mediated cytotoxicity is the release assay. Effector cell-mediated disruption of the target cell membrane results in leakage of its cytoplasmic contents into the culture medium. Endogenous biomolecules (such as lactate dehydrogenase) or previously added exogenous labels (such as the radioisotopes Cr^51^ or In^111^) that leak into the media are then measured as an indirect readout of the damage caused by effector cells. Alternative endpoint methods include flow cytometry, ELISA-based granzyme measurement, and morphometric analyses by microscopy.

Here we describe an automated impedance-based assay that in a real-time and label-free manner captures the kinetics of cell-mediated or antibody-dependent cell-mediated cytolysis (ADCC) of cancer cells. Specifically, the xCELLigence system was used to monitor the response of tumor cells to natural killer (NK) cell activity in the presence or absence of an immunoglobulin G isotype-specific antibody. Importantly, we show that the addition of NK cells in suspension over a monolayer of adherent tumor cells does not cause impedance changes because the NK cells do not come in contact with the electronic sensor. However, in the presence of antibody these non-adherent NK cells inject perforins and granzymes into the tumor cells, causing apoptosis. The resultant morphological changes, death, and detachment of the tumor cells are readily detected via changes in impedance.

Overall, our results show that the impedance-based xCELLigence technology provides a direct, sensitive, and specific measurement of target cell changes over the short (hours) and long (days) time regimes. It enables facile quantification of cell-mediated cytotoxicity and evaluation of the potencies of specific antibodies.

